# Single-Shot 3D Reconstruction via Nonlinear Fringe Transformation: Supervised and Unsupervised Learning Approaches

**DOI:** 10.3390/s24103246

**Published:** 2024-05-20

**Authors:** Andrew-Hieu Nguyen, Zhaoyang Wang

**Affiliations:** 1 Neuroimaging Research Branch, National Institute on Drug Abuse, National Institutes of Health, Baltimore, MD 21224, USA; hieu.nguyen@nih.gov; 2 Department of Mechanical Engineering, School of Engineering, The Catholic University of America, Washington, DC 20064, USA

**Keywords:** fringe projection, deep learning, generative adversarial network, three-dimensional imaging, three-dimensional shape measurement

## Abstract

The field of computer vision has been focusing on achieving accurate three-dimensional (3D) object representations from a single two-dimensional (2D) image through deep artificial neural networks. Recent advancements in 3D shape reconstruction techniques that combine structured light and deep learning show promise in acquiring high-quality geometric information about object surfaces. This paper introduces a new single-shot 3D shape reconstruction method that uses a nonlinear fringe transformation approach through both supervised and unsupervised learning networks. In this method, a deep learning network learns to convert a grayscale fringe input into multiple phase-shifted fringe outputs with different frequencies, which act as an intermediate result for the subsequent 3D reconstruction process using the structured-light fringe projection profilometry technique. Experiments have been conducted to validate the practicality and robustness of the proposed technique. The experimental results demonstrate that the unsupervised learning approach using a deep convolutional generative adversarial network (DCGAN) is superior to the supervised learning approach using UNet in image-to-image generation. The proposed technique’s ability to accurately reconstruct 3D shapes of objects using only a single fringe image opens up vast opportunities for its application across diverse real-world scenarios.

## 1. Introduction

We live in a world where big data are a dominant force, generating an enormous amount of information every second. The capture of comprehensive data from the physical world necessitates the use of a wide range of integrated sensors and system devices, among which cameras are some of the most common. Cameras have a significant impact on many areas of our lives, such as security and surveillance, healthcare and medical imaging, environmental monitoring, aerial photography, and media and entertainment using augmented reality (AR) and virtual reality (VR) [1,2,3,4]. Additionally, cameras play a crucial role in the field of 3D reconstruction, where they are used to capture detailed images and videos of physical spaces or objects, enabling the creation of 3D representations of objects from 2D images.

In the past, achieving both speed and accuracy with high performance in 3D shape reconstruction has been a challenging task due to hardware constraints and algorithm limitations. Various techniques, such as stereo vision, time of flight (ToF), and structure from motion (SfM), prioritize speed over accuracy, making them ideal for application cases that require real-time processing, such as robots, autonomous vehicles, AR/VR, scene understanding, and interactive 3D modeling [5,6,7,8]. Conversely, structured light scanning, digital holography, and patch-based multi-view stereo (PMVS) or photogrammetry are chosen for applications that require good precision and accuracy [9,10,11].

Over the past decade, there has been an ever-growing interest in using artificial intelligence (AI) as a supportive tool for 3D shape reconstruction. With the rapid advancement of deep learning techniques, notably, convolutional neural networks (CNNs) and generative adversarial networks (GANs), researchers have been able to continuously improve and apply these methods within the field of 3D reconstruction. One of the key advantages of employing deep learning models for 3D shape reconstruction lies in their capacity to accurately and efficiently map the intricate structures of physical objects, as captured by 2D imaging, into detailed 3D profiles. This exploit is accomplished through the use of learning-based model networks, which are trained on large datasets comprising conventional 2D images and their corresponding 3D shapes. The integration of 3D reconstruction techniques with deep learning methods mainly depends on the representation of the output. Some common approaches include volumetric representations, surface-based representations, and intermediation techniques [12,13,14,15].

Deep learning has also emerged as a versatile tool in experimental mechanics and optical metrology, offering abilities to enhance the performance of traditional techniques. It has been adopted in a wide range of applications, including defect identification, shape recognition, deformation analysis, quality control, 3D shape reconstruction, stress and strain examination, and fringe pattern and interferogram analysis [16,17,18,19,20,21,22]. Of notable interest is the integration of fringe projection profilometry (FPP) with deep learning, which has attracted significant attention among researchers and engineers. The integration focuses on tasks such as fringe analysis, phase determination, depth estimation, and 3D reconstruction [23,24,25]. While relatively new compared to traditional computer vision techniques, the approach has undergone considerable development and can be classified into various schemes based on the input type (single-shot or multi-shot), stage structure (single-stage or multi-stage), and whether it involves direct conversion or passes through intermediate outputs.

**Figure 1:**
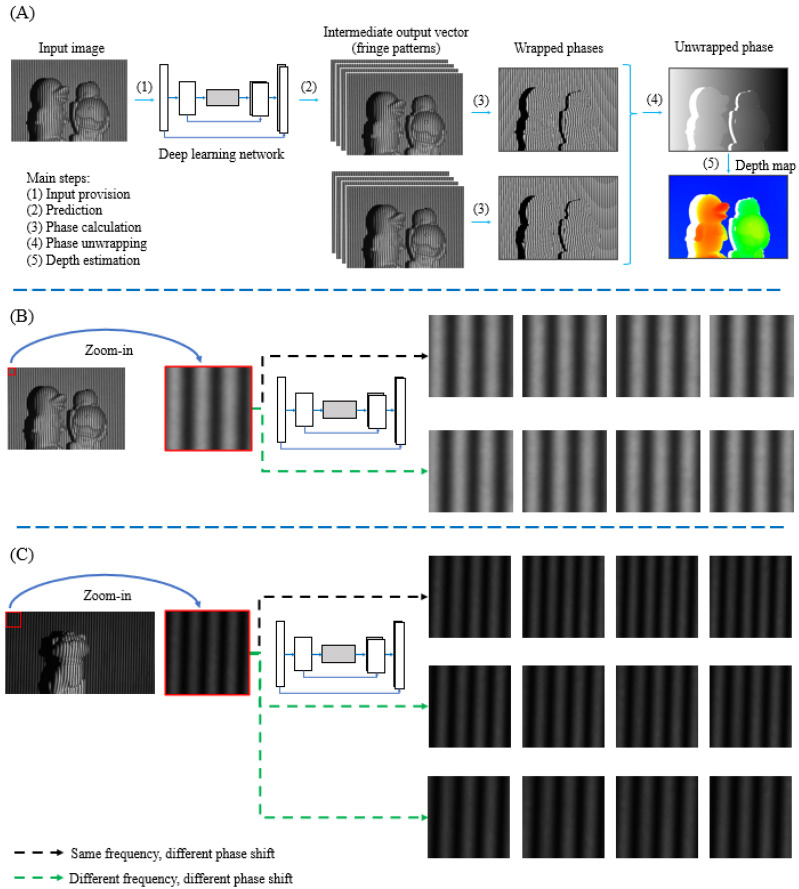
(**A**) Flowchart of the proposed nonlinear fringe transformation for 3D reconstruction; (**B**) dual-frequency nonlinear transformation; (**C**) triple-frequency nonlinear transformation.

This paper introduces an innovative method for reconstructing 3D shape models by integrating fringe projection and deep learning. The proposed approach involves utilizing a deep learning network to perform nonlinear fringe conversion, generating multiple phase-shifted fringe patterns from a single high-frequency fringe image. These predicted fringe patterns are then employed to derive step-by-step intermediate results for classical FPP techniques, such as wrapped phase, fringe order, and unwrapped phase, facilitating subsequent depth estimation and 3D reconstruction. Figure 1A demonstrates a flowchart of the proposed method, describing the step-by-step nonlinear fringe transformation leading to wrapped and unwrapped phase determination and eventual depth estimation.

Classical FPP methods are utilized to gather the necessary datasets to facilitate the learning process using deep learning techniques. Classical FPP techniques within the multi-frequency phase-shifting (MFPS) framework are renowned in optical metrology for their effectiveness in high-accuracy 3D shape reconstruction and deformation determination. This manuscript employs two types of MFPS schemes called the dual-frequency and triple-frequency phase-shifting schemes for training, validation, and testing purposes. The deep learning approach is leveraged to transform a single fringe pattern into multiple fringe patterns at various frequencies and phase shift amounts required for different MFPS schemes. Unsupervised learning with a GAN and a comparable supervised learning network are chosen to evaluate the effectiveness of the proposed methods. To offer a more comprehensive understanding of how the nonlinear fringe transformation operates with varying frequencies and phase shift amounts, Figure 1B,C present an in-depth framework, including a specific zoomed-in region.

The proposed technique is well supported by experimental results and assessments, demonstrating its effectiveness and superiority over existing methods. Its key advantages are as follows:

- Unlike other approaches that require multiple patterns with different frequencies or additional reference images, this technique operates with just a single high-frequency pattern as input.
- It introduces a novel unsupervised learning network (specifically, a GAN) rather than a supervised autoencoder network.
- The nonlinear fringe conversion is achieved through a single network, eliminating the need for multiple sub-networks or a multi-stage network.
- The technique maintains the accuracy advantage of the traditional FPP-based approach while addressing the time-consuming issue associated with acquiring multiple phase-shifted fringe patterns.

This innovative approach holds substantial promise for advancing the 3D imaging and shape reconstruction technology. By providing an accurate and efficient means of generating 3D models for real-world targets, it has the potential to make a notable impact across numerous applications in various fields.

The following parts of the document are organized in the subsequent manner: Section 2 outlines various related methods in the field that integrate fringe projection profilometry technique with deep learning for 3D shape reconstruction. Section 3 depicts the fringe projection method, which includes dual-frequency and triple-frequency approaches. Section 4 describes the structure of the training networks. Section 5 presents diverse quantitative and qualitative outcomes, along with exhibitions of 3D reconstruction. The last two sections discuss potential limitations, applications, future directions, and insights into the overall framework.

## 2. Related Work

The integration of fringe projection with deep learning for 3D reconstruction began in early 2019 with the methods of transforming captured fringe patterns into a depth map [26] or intermediate outputs [27,28,29]. Since then, the integration primarily falls into two categories: direct and indirect methods, regardless of whether the input is a single shot or multiple shots.

The direct method operates on a concept that closely resembles depth estimation or image-to-image conversion in the field of computer vision. In this approach, a network model is trained to transform a single 2D image into either a depth map or an equivalent disparity map. Specifically, the input vector consists of fringe pattern image(s), and the output vector comprises a map image with the same resolution, where the value of each pixel represents geometric depth or height information. Several early methods use autoencoder-based networks, typically UNet, for the fringe-to-depth conversion [20,30], while other approaches use unsupervised network models to predict the depth or height map [31,32]. Nguyen and colleagues enhanced the accuracy of 3D reconstruction by introducing an autoencoder-based network and later incorporating an additional h-shaped global guidance branch in their work [33,34]. Wang et al. proposed SwinConvUNet, which employs a self-attention mechanism and the Swin Transformer for fringe-to-depth conversion, aiming to extract both local and global features [35]. Similar to these works, other researchers [36,37,38,39] presented a few end-to-end network models, such as MSUNet++, PCTNet, DF-Dnet, and DCAHINet; they focus on depth recovery through diverse multi-scale feature fusion modules. The team in [40] introduced LiteF2DNet, a lightweight deep learning framework designed to reduce network weights, and they tested it on computer-aided design (CAD) objects. Further exploration and in-depth adjustment of supervised networks for single-shot 3D measurement led to the development of a depthwise-separable model named DD-Inceptionv2-UNet, as introduced in [41].

In contrast to direct methods, indirect methods encompass various outputs that serve as the link for determining the unwrapped phase map and the final depth map through system calibration. These connecting outputs, often referred to as intermediate outputs, typically originate from the traditional FPP technique. Such outputs consist of phase-shifted fringe patterns, the numerators and denominators of the arctangent function, the wrapped phase map, the unwrapped phase map, and integer fringe orders. In many studies, researchers often convert the input fringe patterns into the numerators and denominators of the arctangent function [42,43,44,45]. Alternatively, transforming the fringe patterns into multiple phase-shifted patterns is an option based on the image-to-image transformation concept [46,47,48,49]. In addition to being predicted through supervised learning, the n-step phase-shifting fringe patterns can also be generated using the virtual temporal phase-shifting method by employing generative adversarial networks (GANs) [50]. However, employing the discrete cosine transform (DCT) for phase unwrapping from a single frequency may result in a less accurate determination of the true phase. The need for employing a multi-frequency phase-shifting approach in the phase unwrapping process has driven the exploration of encoding diverse information into a composite pattern [25,51,52,53,54]. Subsequently, the wrapped phase map can serve as either the input or output vector in cooperation between fringe projection and learning-based networks [55,56,57,58]. Recognizing the significance of the integer fringe orders in the phase unwrapping scheme, several methods have trained CNN models to segment the integer fringe orders or predict the coarse phase map [59,60,61,62]. In recent research, instead of employing multiple networks or a multi-stage scheme to determine separate wrapped phases, fringe orders, or coarse phase maps, a single network with multiple decoder branches has been developed to predict multiple intermediate quantities for determining the unwrapped phase map [63,64,65]. In contrast to simultaneously predicting multiple intermediate outputs, various approaches incorporate multiple input types—such as a reference plane, additional fringe image, intermediate fringe image, wrapped phase, fringe order, etc.—in addition to the fringe pattern, aiming to further improve the accuracy of phase reconstruction [28,66,67,68,69].

## 3. Materials and Methods

The proposed method aims to use both supervised and unsupervised learning networks to transform fringe patterns into desired outputs in a nonlinear manner. The predicted outputs enable the reconstruction of phase information and 3D geometry. To achieve this, an optimized GAN model and a supervised model were trained using datasets with the help of the FPP technique. Dual-frequency and triple-frequency FPP schemes have been adopted for generating the training labels. Details of the FPP technique and the data labeling process are described below.

### 3.1. Fringe Projection Profilometry Technique with Dual-Frequency and Triple-Frequency Four-Step Phase-Shifting Schemes

**Figure 2:**
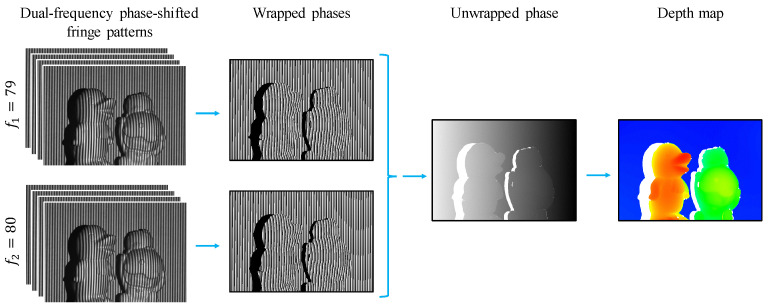
Diagram illustrating the fringe projection profilometry method using a dual-frequency four-step phase-shifting approach.

The FPP method is a widely used technique in experimental mechanics and optical metrology that is capable of measuring 3D shapes with high precision. This approach involves projecting a sequence of fringe images that are phase-shifted onto objects or scenes in a vertical or horizontal direction. The distorted fringe images are captured by a camera, which automatically encodes them with phase and shape information. Figure 2 demonstrates a typical computational workflow of the FPP-based 3D imaging technique.

The fringe patterns captured by the camera can be expressed as follows [29]:

(1) $$I_{i}^{j}\left(u,v\right)=I_{a}\left(u,v\right)+I_{b}\left(u,v\right)\cos\left[\phi_{i}\left(u,v\right)+\delta_{j}\right]$$

where *I*, $I_{a}$, and $I_{b}$ represent the pixel intensities of the captured patterns, the intensity background, and the fringe amplitude at a specific pixel location (u,v). The superscript *j* denotes the order of the phase-shifted image, with *j* ranging from 1 to 4 in the case of a four-step phase-shifting algorithm; the subscript *i* implies the *i*th frequency; δ is the phase-shift amount with $\delta_{j}=\frac{(j-1)\pi }{2}$. The value of $\phi_{i}\left(u,v\right)$ can be computed using the standard four-step phase-shifting algorithm.

The multi-frequency phase-shifting algorithm is commonly used in FPP 3D imaging due to its ability to manage geometric discontinuities and overlapping objects with varying height or depth information. Our proposed approach employs a dual-frequency four-step (DFFS) phase-shifting scheme that uses two fringe frequencies, as well as the triple-frequency four-step (TFFS) scheme, which involves three frequencies. When using the DFFS phase-shifting scheme, the unwrapped phase can be obtained by satisfying the condition that the difference between the two frequencies is one. In such cases, the equations that govern the unwrapped phase can be expressed as follows [52]:

(2) $$\begin{array}{cc} \phi_{12}^{uw} & =\phi_{2}^{w}-\phi_{1}^{w}+\left\{\begin{array}{cc} 0, & \phi_{2}^{w}\geq \phi_{1}^{w}\\ 2\pi , & \phi_{2}^{w}<\phi_{1}^{w} \end{array}\right.\\ \phi & =\phi_{2}^{uw}=\phi_{2}^{w}+\mathrm{INT}\left(\frac{\phi_{12}^{uw}f_{2}-\phi_{2}^{w}}{2\pi }\right)2\pi \end{array}$$

Equation (2) describes the process of unwrapping the phase of two different frequencies, $f_{1}$ and $f_{2}$. It involves using their wrapped phases, $\phi_{1}^{w}$ and $\phi_{2}^{w}$, respectively. However, since the initial unwrapped phase, $\phi_{12}^{uw}$, is derived with only one fringe, it cannot be used directly due to the noise caused by the difference between the two frequencies. Instead, $\phi_{12}^{uw}$ serves as the interfering unwrapped phase for the hierarchical phase-unwrapping process of $\phi_{2}^{uw}$. The final unwrapped phase, denoted as ϕ, corresponds to the phase distribution of the highest fringe frequency. It is noted that this study utilizes $f_{1}=79$ and $f_{2}=80$, which meet the requirements of the DFFS scheme.

The TFFS scheme adopts three fringe frequencies that must meet a specific condition to compute the unwrapped phase of the fringe patterns with the highest frequency. Specifically, it requires that $\left(f_{3}-f_{2}\right)-\left(f_{2}-f_{1}\right)=1$, where $\left(f_{3}-f_{2}\right)>\left(f_{2}-f_{1}\right)>0$. The unwrapped phase can be determined by a set of hierarchical equations [47,53]:

(3) $$\begin{array}{cc} \phi_{12}^{w} & =\phi_{2}^{w}-\phi_{1}^{w}+\left\{\begin{array}{cc} 0 & \;\phi_{2}^{w}⩾\phi_{1}^{w}\\ 2\pi & \;\phi_{2}^{w}<\phi_{1}^{w} \end{array}\right.\\ \phi_{23}^{w} & =\phi_{3}^{w}-\phi_{2}^{w}+\left\{\begin{array}{cc} 0 & \;\phi_{3}^{w}⩾\phi_{2}^{w}\\ 2\pi & \;\phi_{3}^{w}<\phi_{2}^{w} \end{array}\right.\\ \phi_{123} & =\phi_{23}^{w}-\phi_{12}^{w}+\left\{\begin{array}{cc} 0 & \;\phi_{23}^{w}⩾\phi_{12}^{w}\\ 2\pi & \;\phi_{23}^{w}<\phi_{12}^{w} \end{array}\right.\\ \phi_{23} & =\phi_{23}^{w}+\mathrm{INT}\left(\frac{\phi_{123}\left(f_{3}-f_{2}\right)-\phi_{23}^{w}}{2\pi }\right)2\pi \\ \phi & =\phi_{3}^{uw}=\phi_{3}^{w}+\mathrm{INT}\left(\frac{\phi_{23}\frac{f_{3}}{f_{3}-f_{2}}-\phi_{3}^{w}}{2\pi }\right)2\pi \end{array}$$

The conditions for the TFFS scheme may seem more complicated, but they are designed to ensure that the highest-frequency fringe patterns can be accurately analyzed. Equation (3) involves two different types of phases, wrapped and unwrapped, denoted as $\phi^{w}$ and $\phi^{uw}$, respectively. The function “INT” is used to round off numbers to the nearest integer. The term $\phi_{mn}$ represents the difference between the phase values of two points, $\phi_{m}$ and $\phi_{n}$, where the difference $\left(f_{n}-f_{m}\right)$ corresponds to the number of wrapped fringes in the phase map. The algorithm works by using the fact that $\phi_{123}$, which has only one fringe in the pattern, is both wrapped and unwrapped, and this property enables a hierarchical phase-unwrapping process connecting $\phi_{123}$ and $\phi_{3}$ through $\phi_{23}$. Finally, the highest-frequency fringe pattern, $\phi_{3}$, is used for the final phase determination, as it provides the highest level of accuracy.

To extract height or depth information, we utilize the FPP 3D imaging technique, which directly reconstructs the data from the unwrapped phase obtained from Equation (2) or Equation (3). The following explanation of the depth map extraction from ϕ is provided in [70]:

(4) $$\begin{array}{cc} z & =\frac{c{\left[P_{1}\;P_{2}\right]}^{⊺}}{d{\left[P_{1}\;P_{2}\right]}^{⊺}}\\ c & =\left\{1\;\;c_{1}\;\;c_{2}\;\;c_{3}\;\;\cdots \;\;c_{17}\;\;c_{18}\;\;c_{19}\right\}\\ d & =\left\{d_{0}\;\;d_{1}\;\;d_{2}\;\;d_{3}\;\;\cdots \;\;c_{17}\;\;d_{18}\;\;d_{19}\right\}\\ P_{1} & =\left\{1\;\;\phi \;\;u\;\;u\phi \;\;v\;\;v\phi \;\;u^{2}\;\;u^{2}\phi \;\;uv\;\;uv\phi \;\;v^{2}\;\;v^{2}\phi \right\}\\ P_{2} & =\left\{u^{3}\;\;u^{3}\phi \;\;u^{2}v\;\;u^{2}v\phi \;\;uv^{2}\;\;uv^{2}\phi \;\;v^{3}\;\;v^{3}\phi \right\}. \end{array}$$

The parameters, denoted as $c_{1}$ to $c_{19}$ and $d_{0}$ to $d_{19}$ in the equation, are pre-determined through a system calibration process. It is noteworthy that after the determination of the height or depth *z* at each pixel coordinate (u,v), the other two coordinates, *x* and *y*, can be directly determined from the calibrated camera model.

### 3.2. Data Labeling Process

To assess the effectiveness of the proposed nonlinear transformation framework, we employed a 3D camera (Model: RVC-X mini, RVBUST INC., Shenzhen, China) to capture real datasets consisting of a collection of plaster sculptures. The sculptures were chosen for their various geometric surfaces and shapes, with the aim of ensuring that the dataset was diverse and representative of real-world objects. During the capture process, we randomly placed objects in the scene, varying their heights and orientations relative to a reference plane. To further increase the diversity of the dataset, they were grouped in two or more and placed arbitrarily in the scene using the same method but with different orientations and height/depth changes. Typically, objects in the scene were situated within a depth range of −20 mm to 160 mm relative to the reference plane, which was about 850 mm away from the camera baseline.

**Figure 3:**
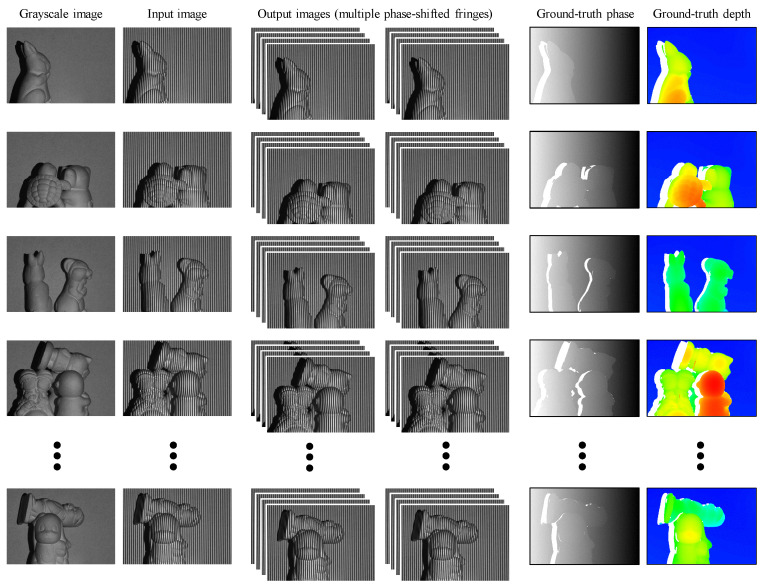
Illustration of the input–output pairs used for training within the datasets employing the DFFS scheme.

Following the described capture strategy, we obtained two separate datasets based on the DFFS and TFFS schemes. The DFFS scheme captured a total of 2048 scenes with a spatial resolution of 640×448. For each scene, we used two different frequencies and a four-step phase-shifting scheme. Each scene was illuminated with eight uniform sinusoidal fringe patterns captured simultaneously by the camera. The first fringe image of each frequency serves as the single input image, denoted as $I_{79}^{1}$ and $I_{80}^{1}$, and all eight captured fringe images form the output vector of the training network. Moreover, a traditional DFFS scheme was used to obtain the ground-truth phase and depth maps for further comparisons. Figure 3 illustrates several examples of the training input–output pairs, as well as the ground-truth unwrapped phase and depth map. Our proposed approach involves taking a single image of a fringe pattern as an input and generating multiple phase-shifted fringe images, as depicted in the second column and the third to fourth columns of Figure 3. Other images, including grayscale images, ground-truth phase maps, and ground-truth depth maps, are included to facilitate comparison of the final 3D reconstruction results.

In addition to the DFFS scheme, we implemented the TFFS scheme using three different fringe frequencies, $f_{1}=61$, $f_{2}=70$, and $f_{3}=80$. This allows for validation of the proposed nonlinear transformation technique with varying spacings in fringe patterns. The TFFS datasets comprise a total of 1500 data samples, each with a resolution of 640×352, and 12 fringe images were recorded for each scene.

## 4. Supervised and Unsupervised Networks for Nonlinear Fringe Transformation

### 4.1. Network Architecture

**Figure 4:**
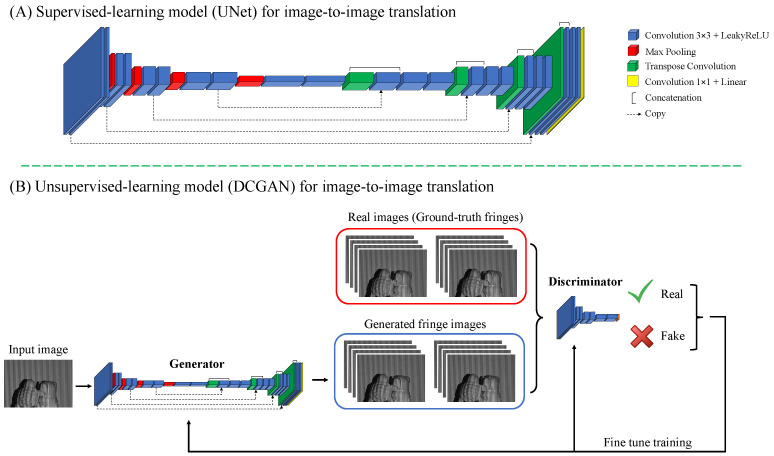
Architecture of the supervised UNet (**A**) and unsupervised DCGAN (**B**) models utilized in the training process.

Our proposed work achieves image-to-image translation through a supervised learning approach utilizing the UNet architecture, a widely recognized autoencoder-based network specifically tailored for image segmentation tasks. The UNet architecture comprises an encoder and a decoder path, forming a U-shaped structure that includes a bottleneck at the bottom connecting the encoder and decoder. The encoder path gradually reduces the spatial dimension through max-pooling layers, while filter sizes are increased to extract hierarchical features using convolution layers. The decoder path reverses these operations using transposed convolutions for up-sampling. To preserve fine feature information during up-sampling, skip connections are utilized to copy corresponding layers from the encoder and concatenate them with the decoder path. Each convolution layer within the UNet architecture incorporates a LeakyReLU activation function with an alpha value set to 0.2, effectively addressing the zero-gradient problem commonly encountered in training deep neural networks. The final stage of the process involves linear activation through a 1×1 convolution layer, which maps the extracted features to arbitrary pixel values necessary for generating fringe patterns. For further insights into the UNet model’s application in fringe-to-fringe transformation, detailed information can be found in Refs. [47,52]. Figure 4A shows the supervised UNet model tailored for nonlinear fringe transformation tasks.

In unsupervised learning, the image generation task involves using a deep convolutional generative adversarial network (DCGAN) for nonlinear fringe generation based on different-frequency fringe input. The DCGAN consists of a generator and a discriminator, both trained simultaneously through adversarial training. The generator’s objective is to create realistic fringes that can deceive the discriminator, while the discriminator aims to accurately distinguish between real and generated/fake fringes. Through the adversarial training process involving generator and discriminator loss functions, the weights are updated, encouraging the generator to progressively enhance its ability to produce lifelike fringes. Figure 4B visualizes the architecture of the unsupervised model DCGAN, which includes two separate parts of the generator and discriminator.

The generator’s architecture is similar to that of the UNet model mentioned earlier, with the objective of generating fringe patterns closely resembling the ground-truth fringes (real fringes). The generator comprises a series of transposed convolutional layers that up-sample the input noise vector to generate the final output images. The generator also contains skip connections that copy the corresponding layers from the encoder, concatenating them with the decoder path to preserve fine feature information during upsampling. The discriminator, on the other hand, takes both generated and real fringes as input vectors, discerning between them. The discriminator incorporates convolution layers with a stride of 2 to extract feature information from the input feature map. Each convolution layer undergoes batch normalization and applies the LeakyReLU activation function to introduce nonlinearity. The final output layer uses a sigmoid activation function for binary cross-entropy loss, generating a probability score that indicates the likelihood that the input image is either real or generated/fake. In particular, probability scores approaching 1 indicate real fringes, while values approaching 0 signify generated fringes. The adversarial training process between the generator and discriminator continues until the generator can produce lifelike fringes that deceive the discriminator into believing they are real.

### 4.2. Hyperparameter Tuning and Training Process

The process of capturing and processing image datasets requires a great deal of effort and attention. In this case, an RVC-X camera was used to capture the raw images, followed by a pre-processing step to prepare the input–output pair for the training process. To ensure that the input images are in an appropriate format, the input fringe images undergo a normalization to a scale of [−1,1]. Meanwhile, the output images could either be in the raw format combined with a linear activation function or scaled down to [−1,1] with the tanh activation function. This step is essential to ensure that the input and output images are comparable and that the neural network can accurately learn the relationship between them.

Supervised learning involves partitioning the entire dataset into three sets—the training set, the validation set, and the test set. In this work, the partitioning is 80%:10%:10%. A Keras framework handles the internal split between the training and validation sets. During the supervised learning phase, the Adam optimizer was employed with an initial learning rate of 0.0001 for 300 learning epochs. After that, a step decay schedule was utilized for the last 100 epochs to gradually diminish the learning rate, aiding in the convergence of the network. The batch size was set to 1, and the loss function for the image regression task is the mean squared error (MSE).

In the unsupervised learning scenario, the dataset was divided into training and test sets using a ratio of 90%:10%. The Adam optimizer, with a learning rate of 0.0002 and a beta value of 0.5, was applied to both the discriminator and the entire DCGAN model. The discriminator utilized the MSE as the loss function, focusing on distinguishing real fringes from the generated ones. The DCGAN loss is a combination of sigmoid cross-entropy and L1 loss (mean absolute error—MAE), with a weight loss ratio of 1 versus 100 (i.e., LAMBDA = 100). PatchGAN, a typical discriminator architecture for image-to-image translation, was employed to discern real fringes or generated fringes at the patch level, using arrays of 1’s and 0’s instead of a single output, as seen in traditional discriminators. The entire learning process involves 200 iterative epochs with a batch size of 2. Notably, samples are randomly selected internally in each epoch. In both the supervised and unsupervised learning processes, a 10% split test set is completely separated from the training and validation datasets. This ensures that the object surface has not been encountered during training or validation, thereby mitigating possible overfitting issues and biased evaluations.

Multiple graphics processing unit (GPU) nodes within the Biowulf cluster at the National Institutes of Health (NIH) were required to train the models. Specifically, the two main GPU nodes utilized in this project consist of 4 × NVIDIA A100 GPUs with 80 GB VRAM each and 4 × NVIDIA V100-SXM2 GPUs with 32GB VRAM each. The programming framework relies on Python 3.10.8, and the deep learning Keras framework version 2.11.0 is employed to construct the network architecture, as well as for data prediction and analysis. It is worth noting that the training time for the DCGAN model was 9 h using NVIDIA A100 GPUs with a batch size of 2 and 200 epochs. In contrast, the UNet model required less than 6 h for 400 epochs. This highlights the importance of choosing the right model architecture and training parameters to optimize the training time without compromising the quality of the results.

## 5. Experiments and Results

### 5.1. Assessment of Image Quality for Generated Fringe Patterns

Our proposed approach for achieving accurate 3D reconstruction begins with a nonlinear fringe transformation utilizing cutting-edge image-to-image conversion techniques. To ensure visual fidelity in the predicted fringe patterns, we employ a range of robust image quality assessment methods. Notably, we use the structural similarity index measurement (SSIM) and peak signal-to-noise ratio (PSNR) to assess the quality disparity between real and generated fringes. Through this methodology, we are assured of our ability to deliver the most accurate and reliable 3D reconstructions possible.

**Figure 5:**
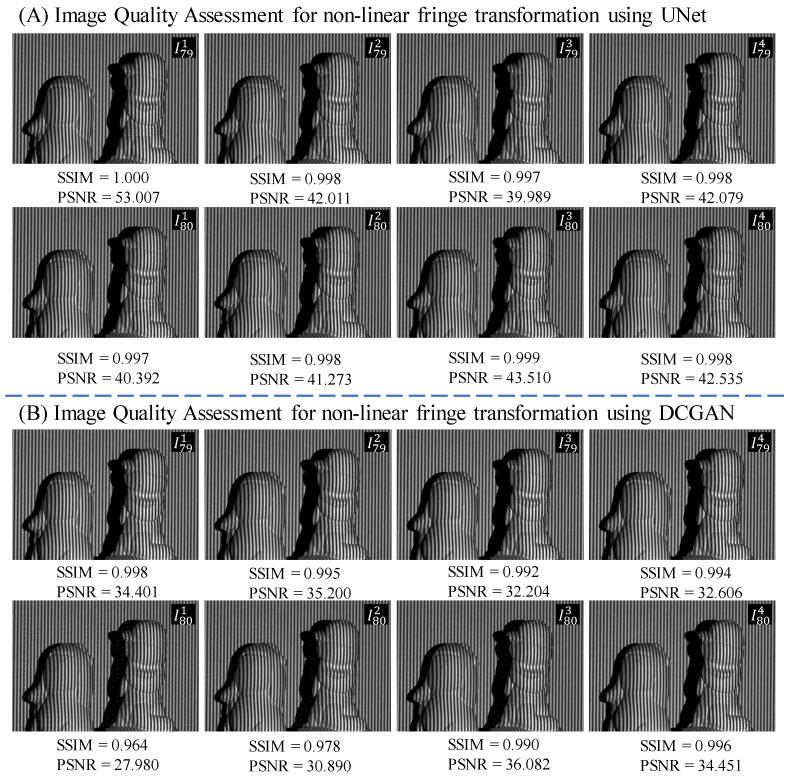
Assessment of image quality using the SSIM and PSNR metrics for predicted fringe images.

This experiment evaluates the image quality and quantitative metrics of generated fringe patterns using two deep neural network models, specifically, the UNet and DCGAN. Figure 5 illustrates a comparative analysis of the performance of these models. The UNet model exhibited superior performance by accurately producing the initial four fringes of $I_{79}^{1-4}$ with high SSIM scores ranging from 0.998 to 1.000 and PSNR values exceeding 40. This success was attributed to the selection of an appropriate fringe input, $I_{79}^{1}$, and the network’s ability to accurately shift the fringe patterns by a phase shift amount of ϕ=π/2. The initial predicted fringe closely resembled the input, resulting in the highest SSIM and PSNR scores. Furthermore, the UNet model effectively predicted the subsequent four fringes with a different frequency of $f_{2}=80$, representing a nonlinear fringe transformation task where the number of fringes in the generated images increased by one fringe and images underwent a phase shift of ϕ=π/2. Despite its relatively lower SSIM and PSNR values, the UNet model demonstrated its ability to generate satisfactory fringe patterns for the final 3D reconstruction process.

In comparison, the DCGAN model demonstrated an inferior performance to its supervised UNet counterpart. The eight generated fringes and their corresponding SSIM and PSNR values indicated a decline in quality compared to the real fringes. The initial four fringes at the same frequency $f_{1}=79$ as the fringe input were predicted correctly, but the subsequent four fringes at frequency $f_{2}=80$ showed a deterioration in quality compared to the real fringes. Notably, the degradation of the four fringes $I_{80}^{1-4}$ was evident in the last row, where several vertical white pixels were visible.

### 5.2. The 3D Reconstruction of a Single Object and Multiple Objects via the DFFS Scheme

**Figure 6:**
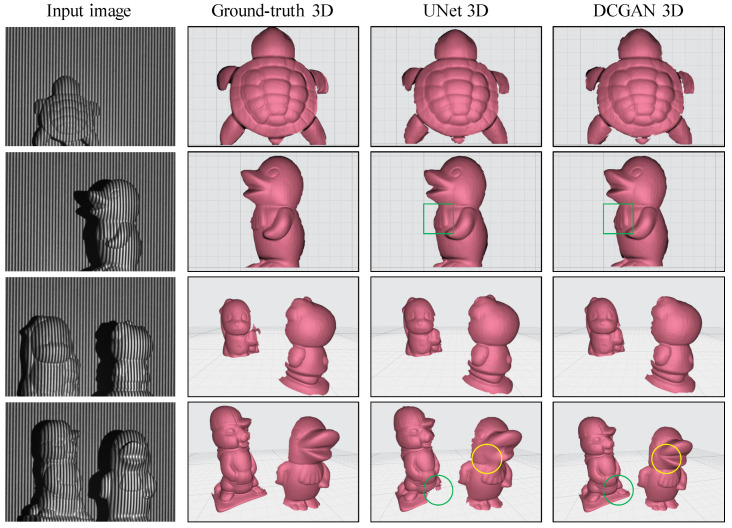
Comparison of the 3D reconstruction results between UNet and DCGAN models when utilizing the DFFS scheme.

After successfully training the model, we proceeded to utilize a single fringe to generate new fringe patterns. The experiment employed the DFFS scheme with $I_{79}^{1}$ as the initial fringe input. The trained model produced eight fringes of $I_{79}^{1-4}$ and $I_{80}^{1-4}$ as outputs. Figure 6 shows a few representative results obtained by using two different approaches: UNet and DCGAN. The first column in the figure presents the fringe input to the network, followed by the ground-truth 3D shape generated by the conventional DFFS scheme. Subsequently, the third and fourth columns display the 3D reconstruction, highlighting key deviations from the reference 3D shape.

To evaluate the model’s performance, we conducted a comparative analysis between the UNet and DCGAN schemes. While both approaches successfully rendered an overall 3D representation of the targets, distinctions in their performance emerged. Upon closer examination of the generated 3D shapes, it became evident that the DCGAN model produced more detailed depth information than the UNet did, as the region highlighted by a green square shows. Moreover, the region highlighted by a green circle indicated that the UNet model struggled to generate the desired 3D shape due to incorrect fringe orders. The region in the yellow circle further shows the UNet’s deficiency in capturing certain depth details owing to inaccuracies in fringe ordering. Additionally, we noted some shape discontinuities along the edges for both approaches.

### 5.3. Investigation of the Frequency-Dependent Fringe Input of $I_{79}^{1}$ and $I_{80}^{1}$

**Figure 7:**
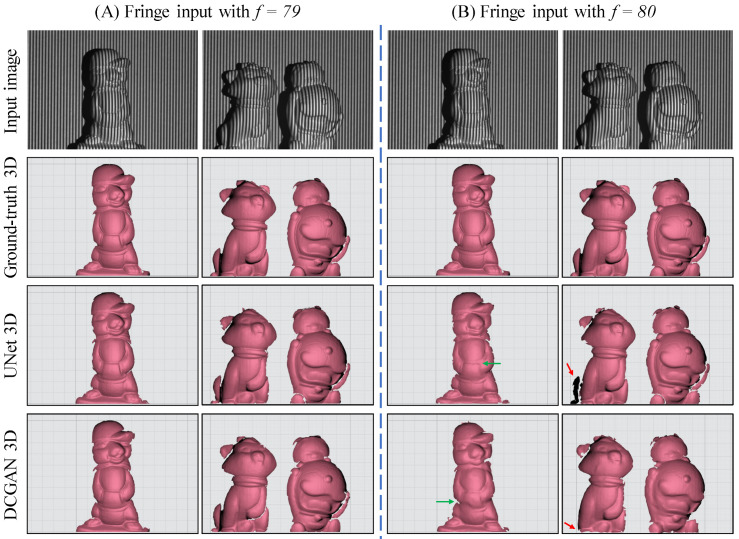
Comparison of 3D reconstruction using distinct fringe inputs at frequencies f=79 and f=80.

There is a growing concern about how using different fringe inputs with different frequencies could affect the reconstruction of 3D shapes. To investigate this issue, two additional networks, UNet and DCGAN, were trained using one fringe input $I_{80}^{1}$ while keeping the output fringes as either $I_{79}^{1-4}$ or $I_{80}^{1-4}$. Figure 7 is divided into two sections to make it easier to visually compare the use of different fringe inputs.

Based on the visual observations, it appears that the performance of both the UNet and DCGAN models is inferior when using the fringe input $I_{80}^{1}$ in comparison with $I_{79}^{1}$. This discrepancy arises from the surfaces of the shapes appearing blurred due to subsequent post-processing steps aimed at filling gaps and eliminating shape irregularities. It is important to note that these post-processing steps are applied uniformly to all reconstruction regions with identical parameters to ensure a fair visual comparison. For a more detailed examination of single-object reconstruction, the green arrows highlight the rough nature of the reconstructions and their inability to capture finer details when the network operates with a fringe frequency of f=80. Furthermore, the red arrows point out shape-disconnected regions and missing areas in the reconstructed object when using the fringe input $I_{80}^{1}$.

Overall, the observations indicate that employing the fringe input $I_{80}^{1}$ leads to inferior shape reconstructions characterized by disconnected regions and a deficiency in fine details. Conversely, utilizing the fringe input $I_{79}^{1}$ yields a clearer and more accurate shape representation.

### 5.4. The 3D Reconstruction of a Single-Object Scene via the TFFS Scheme

The previous tests were carried out using the DFFS scheme, which involves a nonlinear fringe transformation to generate fringe patterns with two distinct frequencies, and the frequency difference is exactly one. However, it is unclear if deep learning networks can effortlessly handle multiple frequencies. Therefore, this experiment uses the TFFS scheme to perform a more complex nonlinear transformation task. In the TFFS scheme, the differences in fringe frequencies are much larger; specifically, the following three frequencies are used: $f_{1}=61$, $f_{2}=70$, and $f_{3}=80$. In this particular experiment, the fringe input is $I_{80}^{1}$ and the output arrays consist of 12 fringes, namely, $I_{61}^{1-4}$, $I_{70}^{1-4}$, and $I_{80}^{1-4}$.

**Figure 8:**
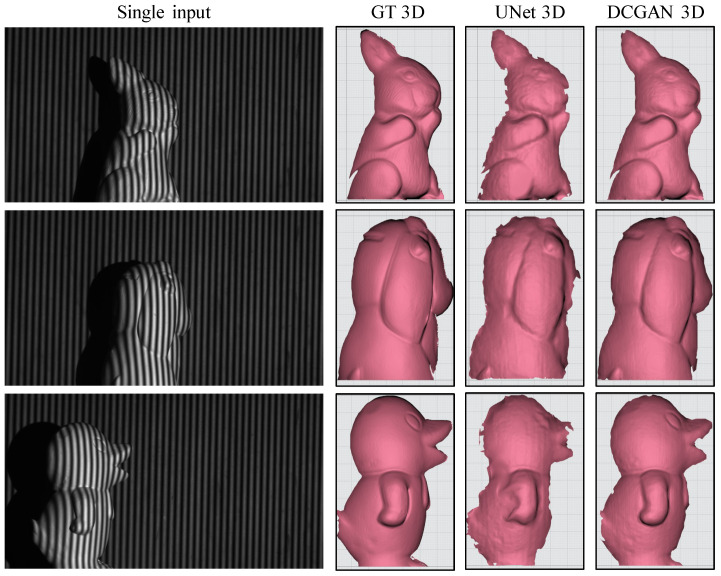
Visualization of 3D reconstruction in single-object scenes utilizing the TFFS scheme.

The results of 3D reconstruction using the TFFS scheme are presented in Figure 8. Again, a comparison between the UNet and DCGAN models for nonlinear fringe transformation is conducted. Upon analysis, it is evident that the unsupervised learning approach utilized by the DCGAN model outperforms the UNet model.

The supervised learning approach is limited in its ability to capture objects’ shape details accurately, and it introduces considerable noise and discontinuities along the edges. In contrast, the unsupervised learning approach employed by DCGAN excels in reconstructing most of the shape details, resembling the ground-truth 3D shape with a higher accuracy. The DCGAN model’s ability to learn from unlabelled data is the key to its superior performance, as it can capture more complex features and patterns in the object’s shape, leading to higher accuracy and better reconstruction results.

## 6. Discussion

This article presents an innovative method for reconstructing 3D image(s) by integrating structured light and deep learning. The proposed approach involves training a single fringe pattern to generate multiple phase-shifted patterns with varying frequencies, followed by a conventional algorithm for subsequent 3D reconstruction. Validation of the technique was conducted on two datasets, employing both supervised and unsupervised deep learning networks to execute the nonlinear fringe transformation. The results showed that the unsupervised learning strategy using the DCGAN model outperformed the supervised UNet model on both datasets.

During the training and prediction stages, both supervised and unsupervised learning models tend to produce some incorrect fringe orders during obtaining the unwrapped phase. These errors lead to the generation of multiple layers of 3D shapes and irrelevant scattering point clouds. However, these limitations can be addressed with various automatic noise-removing techniques. It is noteworthy that prior state-of-the-art techniques, which had sufficient input information, such as encoded composite fringes, multiple fringes, or fringes with a reference image, did not encounter such issues. Nevertheless, the elimination of irrelevant point clouds can also be achieved manually through post-processing steps. The comparison between UNet and DCGAN also highlighted the importance of obtaining accurate fringe orders in generating detailed 3D shapes, which is an obvious requirement. It is suggested that further exploration in this area could lead to more precise 3D reconstruction results.

In our proposed approach, we utilized the DFFS and TFFS schemes with high-frequency fringe patterns for both training and testing scenarios. The use of low-frequency (e.g., 4, 8, etc.) fringe images is undesirable, as they typically result in lower accuracy in phase determinations. As demonstrated in our previous work, low-frequency fringe patterns yield inferior 3D reconstruction results compared to high-frequency fringe patterns in the image-to-depth construction process [34].

This study used both an unsupervised DCGAN and the conditional pix2pix model [71] for image-to-image translation and compared their performance. Pix2pix is a type of conditional GAN where the input is combined with both real and generated images to distinguish between real and fake. However, the pix2pix model was observed to be less accurate in generating the 3D shape of the object and introduced more scattered noise and incorrect layers. It should be noted that the pix2pix model was trained in parallel with the DCGAN model, but none of its results outperformed the UNet and DCGAN models. Therefore, we have left out the relevant descriptions. It is also noted that the DCGAN yields some unexpected artifacts, which can be seen in Figure 5. Although such artifacts are common in GAN models, our experimental results have demonstrated that these generative traits effectively cope with the nonlinear fringe transformation task, whereas the supervised learning model falls short.

Given that the proposed networks are trained on robust GPU cards, i.e., NVIDIA A100 and V100-SXM2, equipped with lots of VRAM, the likelihood of encountering out-of-memory issues during training is significantly reduced. However, rather than generating multiple intermediate outputs in the form of phase-shifted fringe patterns, a more efficient strategy could be producing the numerators and denominators used in subsequent phase calculation. This approach can help cope with the potential memory-related challenges. In this specific situation, the memory required for the outputs can be reduced to half. It is worth mentioning that training for 400 epochs in the supervised learning process is significantly faster than training for 200 epochs in the unsupervised learning approach. This discrepancy arises because the supervised learning approach loads all of the training and validation datasets into the VRAM at once and conducts internal splitting, whereas the unsupervised learning approach automatically draws a small batch randomly into the VRAM for training. Additionally, the inclusion of an additional discriminator architecture, along with the fine-tuning process, adds to the computational cost of the unsupervised learning approach.

We acknowledge that this paper lacks a thorough comparison with other cutting-edge techniques for 3D reconstruction that integrate fringe projection and deep learning. Obtaining sample codes from these techniques is challenging, and each method involves its own hyperparameter tuning and network construction. Consequently, the proposed approach only utilizes the most popular network for fringe-to-fringe transformation, specifically, an autoencoder-based UNet, to compare it with the unsupervised-learning-based DCGAN. Furthermore, the proposed technique primarily serves as a proof of concept for nonlinear fringe transformation using deep learning. More comprehensive comparisons can be performed in future work as the field progresses. Future research could explore more rigorous comparisons and strive to enhance the accuracy of the nonlinear fringe transformation method.

Given the inherent complexity in constructing robust network architectures and tuning deep learning models, we recognize that future datasets should include a wider variety of objects and more challenging scenes with diverse testing conditions to address the concern of possible overfitting issues. We are committed to expanding our dataset collection efforts to provide the research community with more comprehensive and representative datasets.

## 7. Conclusions

In summary, this manuscript introduces a novel 3D reconstruction approach via a nonlinear fringe transformation method that combines fringe projection with deep learning. This technique utilizes a single high-frequency fringe image as input and generates multiple high-frequency fringe images with varying frequencies and phase shift amounts. These intermediate outputs facilitate the subsequent 3D reconstruction process through the traditional FPP technique. Since the proposed method requires only a single fringe image to reconstruct the 3D shapes of objects with good accuracy, it provides great potential for various dynamic applications, such as 3D body scanning, heritage and preservation scanning, virtual clothing try-ons, indoor mapping, and beyond. Regarding future works, there is a wide range of possibilities for exploring and enhancing the proposed approach. One possible direction is to investigate the method’s performance on larger datasets and more complex objects. Moreover, researchers can explore different imaging configurations to enhance reconstruction quality and accuracy. Additionally, the potential of this method is worth exploring in other domains, such as medical imaging or robotics.

## Data Availability

The data presented in this study are available upon request from the corresponding author.
